# Hurry Up, We Need to Find the Key! How Regulatory Focus Design Affects Children’s Trust in a Social Robot

**DOI:** 10.3389/frobt.2021.652035

**Published:** 2021-07-08

**Authors:** Natalia Calvo-Barajas, Maha Elgarf, Giulia Perugia, Ana Paiva, Christopher Peters, Ginevra Castellano

**Affiliations:** ^1^ Uppsala Social Robotics Lab, Department of Information Technology, Uppsala University, Uppsala, Sweden; ^2^ Embodied Social Agents Lab (ESAL), School of Electrical Engineering and Computer Science, KTH Royal Institute of Technology, Stockholm, Sweden; ^3^ Department of Computer Science and Engineering, Instituto Superior Técnico (IST), University of Lisbon, Lisbon, Portugal

**Keywords:** trust, child–robot interaction, regulatory focus, goal orientation, affective, emotional robot

## Abstract

In educational scenarios involving social robots, understanding the way robot behaviors affect children’s motivation to achieve their learning goals is of vital importance. It is crucial for the formation of a trust relationship between the child and the robot so that the robot can effectively fulfill its role as a learning companion. In this study, we investigate the effect of a regulatory focus design scenario on the way children interact with a social robot. Regulatory focus theory is a type of self-regulation that involves specific strategies in pursuit of goals. It provides insights into how a person achieves a particular goal, either through a strategy focused on “promotion” that aims to achieve positive outcomes or through one focused on “prevention” that aims to avoid negative outcomes. In a user study, 69 children (7–9 years old) played a regulatory focus design goal-oriented collaborative game with the EMYS robot. We assessed children’s perception of likability and competence and their trust in the robot, as well as their willingness to follow the robot’s suggestions when pursuing a goal. Results showed that children perceived the prevention-focused robot as being more likable than the promotion-focused robot. We observed that a regulatory focus design did not directly affect trust. However, the perception of likability and competence was positively correlated with children’s trust but negatively correlated with children’s acceptance of the robot’s suggestions.

## 1 Introduction

Nowadays, social robots are becoming more popular in fields such as healthcare (Dawe et al., 2019), education (Leite et al., 2014), and assistive therapy (Perugia et al., 2020). In educational settings, for example, social robots have been proven successful in offering socially supportive behaviors (e.g., nonverbal feedback, attention guiding, and scaffolding) that not only benefit children’s learning goals (Saerbeck et al., 2010; Belpaeme et al., 2018) but are also associated with relationship formation and trust development during the interaction (van Straten et al., 2020; Chen et al., 2020).

Robots in education are used as companions to support children in a large variety of subjects and tasks (Leite et al., 2015; Westlund et al., 2017; Gordon et al., 2016). A review on social robots in education pointed out that personalized robots lead to greater affective (i.e., receptiveness, responsiveness, attention, and reflectiveness) and cognitive (i.e., knowledge, comprehension, analysis, and evaluation) learning gains in scenarios where the robot acts as a tutor providing curriculum support and supervision or as a peer and learning companion (Belpaeme et al., 2018). Hence, to ensure a constructive child–robot schooling experience, educational robots should be designed to give customized support so as to achieve higher performance from students at pursuing their goals.

As such, it is crucial to establish what verbal and nonverbal behaviors robots can use to increase children’s learning, engagement, and trust in the robot. One way to understand the effect of the robot’s behaviors on children’s affective and cognitive learning gains is by investigating child–robot relationship formation. The literature in social psychology suggests that teachers’ social skills (e.g., nonverbal behavior, communication strategies, and the way they interact with learners) foster more trusting child–teacher relationships that are crucial for children’s performance (Witt et al., 2004; Howes and Ritchie, 2002). For instance, students’ interest toward academic and social goal pursuit is encouraged by teachers who give positive feedback (Ryan and Grolnick, 1986). There is evidence that children who do not trust their tutors or teachers are unable to use them as a resource for learning but also that the lack of trust makes the child–teacher relationship difficult (Howes and Ritchie, 2002). Therefore, teachers’ behavior should promote emotional and social support to facilitate a trustworthy child–teacher relationship.

Also, in child–robot interaction (cHRI), several studies have investigated the way the robot’s behaviors and actions can support interactions to meet the children’s needs (Saerbeck et al., 2010; Leite et al., 2014). During this process, building a trusting child–robot relationship is crucial. Once children trust the robot, they will use it to structure their learning, as the robot is designed to attend to their comments, provide help, or give positive feedback to their discoveries (Kahn et al., 2012; Tielman et al., 2014; Vogt et al., 2019). Therefore, the initial step is to investigate how children build a trust model of a robot. In this study, we focus on understanding if and how the robot’s behaviors affect children’s perceptions of its trustworthiness in a goal-oriented activity.

During goal pursuit, the regulatory focus theory (RFT) introduces the principle that individuals guide their actions by adopting one of two self-regulatory strategies: promotion and prevention (Higgins, 1997; Higgins, 1998). For example, if the goal is to qualify for the finals of a tournament, a promotion-focused person will train extra hours with the aim of winning the tournament, while a prevention-focused person will train just enough to avoid failing the qualification. These strategies are related to the motivational orientation people have to achieve their goals. Whereas individuals in a promotion focus are eager to attain advances and gains, individuals in a prevention focus are vigilant to ensure safety and avoid losses. As such, RFT has been found to positively impact creativity (Baas et al., 2008) and idea generation (Beuk and Basadur, 2016) and to induce longer social engagement (Agrigoroaie et al., 2020).

Regarding the application of RFT in human–robot Interaction (HRI), the literature is scarce and limited to adults. Most of the available studies investigated how RFT can be used to adapt the robot’s behaviors to the user’s state (Cruz-Maya et al., 2017; Agrigoroaie et al., 2020). This adaptation is carried out by matching the robot’s regulatory focus personality type to the user’s regulatory focus orientation, which is known as the regulatory fit theory (Higgins, 2005).

The RFT has not been investigated before in cHRI. Therefore, there is no evidence yet of its effects on children’s performance in a goal-oriented activity and its relationship with children’s trust and the robot’s likability. Our research study is the first work in cHRI to investigate whether RFT can be effectively applied to the design of the whole interaction rather than only to the robot’s personality (i.e., matching the robot’s behavior to the child’s regulatory focus type). Within an educational context, we aim at investigating the possible effects of regulatory focus designs on emotional induction and engagement (Elgarf et al., 2021), narrative creativity and learning, and child–robot relationship formation. In this context, the present contribution focuses on assessing whether RFT can be used as a design strategy that promotes trust development between a child and a robot. Thus, we designed an educational scenario where an EMYS robot plays the role of a companion that guides and supports the child through an interactive collaborative game.

The main research question we address is *whether a regulatory focus design scenario has an effect on the way children interact with the robot and, specifically, on their perceptions of the robot*’*s trustworthiness and reliance on the robot*. To investigate this question, two versions of the game were created following two different self-regulation strategies: 1) a prevention-focused game, where the robot engages in the activity with the goal of avoiding a risk and 2) a promotion-focused game, where the goal is seeking a reward. Results show that a regulatory focus design scenario influences children’s perceptions of the likability of the robot. It does not directly affect the way in which children create a trust model of a social robot but does so indirectly through the mediation of perceived likability and competence. These results are important for the HRI community as they provide new insights into the effects of a robot’s educational strategies on children’s perception of its trustworthiness.

## 2 Related Work

### 2.1 Regulatory Focus Theory

The RFT, introduced by Higgins (1997, 1998), explains that people adopt one of two possible approaches when pursuing goals: promotion and prevention. In a promotion focus, individuals focus their attention on attaining positive outcomes (e.g., excitement and happiness) which are related to the importance of fulfilling goals and aspirations (i.e., achieving goal motivation). In a prevention focus, people aim at avoiding negative outcomes (e.g., stress and anxiety) which are linked to the importance of ensuring safety and being responsible (i.e., avoiding failure motivation) (Higgins, 1998). Furthermore, the literature suggested that RFT affects individuals’ attitudes and behaviors (Higgins and Cornwell, 2016). An example is given in the study by Beuk and Basadur (2016), who found that promotion focus had a positive effect on task engagement.

RFT may also be beneficial in a variety of disciplines. For instance, Liberman et al. (2001) found that undergraduate students with a promotion focus developed more solutions for problems than students with a prevention focus. Another example is the impact of RFT on creativity. Friedman and Förster (2001) investigated the effect of approach-avoidance motivation on individuals who engaged in a creativity task. To do so, participants were primed with a task to manipulate RFT. The task consisted of a mouse trapped in a maze, and participants needed to find a way to get the mouse out of the maze. In the promotion focus, a piece of cheese (gain) was lying outside the maze, whereas in the prevention focus, there was an owl (threat). The authors found that the promotion-focused orientation fostered creative insight and divergent thinking, compared to the prevention-focused orientation. A recent study confirmed this result, showing that promotion-focused orientation significantly impacted the quantity and type of ideas generated by individuals who participated in a divergent thinking task (Beuk and Basadur, 2016).

Besides, recent studies have demonstrated that in social interactions, this type of self-regulation influences individuals’ trust perception. Keller et al. (2015) found that the prevention focus lowered individuals’ generalized trust in a trust game paradigm. The authors suggested that prevention-focused regulation is associated with a need for security and a vigilant tendency to avoid losses or negative events, and therefore, affects people’s willingness to trust others in social interactions that entail threats. Another study found that regulatory focus can also influence an individual’s degree of endorsement and reliance when making decisions (Cornwell and Higgins, 2016). A recent research study investigated how priming participants with a prevention focus induces less trust than priming them with a promotion focus in a trust game when goals are not fulfilled.

In HRI, the study of RFT is in its early days and has not received enough attention. Recent studies have investigated how a regulatory focus type robot (promotion and prevention) affects the user’s performance. These studies presented the effects of matching the behavior of the robot with the participants’ regulatory focus type (also known as regulatory fit theory) (Higgins, 2005). In the study by Cruz-Maya et al. (2017), individuals who interacted with a regulatory focus–oriented robot had a better performance in a Stroop test. A follow-up study showed how a robot persuaded participants more in a collaborative game when it tailored its behavior to the users’ regulatory focus orientation (Cruz-Maya and Tapus, 2018). In another study, a robot that displayed promotion and prevention behaviors encouraged participants to engage in longer interactions (Agrigoroaie et al., 2020). Also, RFT has been investigated in virtual agents. Faur et al. (2015) found that individuals with a prevention focus orientation liked the agent more than individuals with a promotion focus. As far as HRI is concerned, there is evidence on adults that indicates that promotion focus regulation is positively correlated with an increment of a robot’s persuasiveness when pursuing a goal (Cruz-Maya and Tapus, 2018).

No previous work has studied regulatory focus design and its effects on trust or relationship formation in HRI or cHRI. However, there is evidence that the robot’s design can prime and induce users to a certain level of trust (Kok and Soh, 2020). Moreover, due to the fact that RFT originates from distinct survival needs, regulatory focus design might have significant implications with regard to trust perception and relationship formation that are worth exploring. To the best of our knowledge, this is the first experimental study that uses RFT to design a goal-oriented activity for cHRI in an educational scenario.

### 2.2 Trust in cHRI

Trust is a complex and multifaceted phenomenon which requires special attention for its investigation. Within psychology, trust can be defined and measured along two main dimensions: affect- and cognition-based trust. The first encompasses interpersonal trust (e.g., benevolence, interpersonal care, sincerity, and perceived warmth), while the second assesses perceived competence, ability, and reliability (McAllister, 1995; Kim et al., 2009). Children’s trust is assessed by using multi-methodological approaches aimed at investigating the role of trust in children’s social and intellectual development (Bernath and Feshbach, 1995). Research in psychology has investigated the role of friendship to explore children’s trust conceptions and judgments. These studies suggested that peer trust influences the social acceptance that promotes trust development (Bernath and Feshbach, 1995). A recent study found that children evaluate competence and benevolence differently and use this judgment as a source of information to determine whom to trust (Johnston et al., 2015).

This distinction between affect- and cognition-based trust has been examined in HRI. In a recent study, Malle and Ullman (2021) argued that robots have been introduced as social agents that are evaluated for their performance (ability and reliability) but also for their moral characteristics (sincerity and integrity). An example of this is given in the study by Cameron et al. (2020), who investigated how the robot’s behaviors affect the user’s perception of trust. They found that a robot that discloses its mistakes and tries to rectify a faulty situation is perceived as more capable and trustworthy but less likable than a robot that only recognizes its errors.

There is some evidence in regard to conceptualizing the multifaceted nature of trust in cHRI. van Straten et al. (2018) found that children differentiate between interpersonal and technological trust when making judgments about the trustworthiness of social robots. Stower et al. (2021) conducted a meta-analysis of robot-related factors (i.e., embodiment and behaviors) that have been identified as influencing trust in cHRI. To do so, the authors distinguished between two domains for children’s trust in social robots: social trust, defined as the “belief that the robot will keep its word or promises,” and competency trust, defined as “perceived competency and reliability of the robot.” From 20 studies, they found that a social robot that exhibits more humanlike attributes does not always lead to a higher competency trust and liking. Also, they found that the type of measure used to capture children’s social trust in robots influences the direction of the effect.

Recent cHRI studies have dealt with the design of the robot’s behaviors to assess children’s trust in robots. Kennedy et al. (2015) found that a contingent robot increased children’s compliance with the robot’s suggestions and therefore elicited higher competency trust in the robot. In another study, children trusted and liked a contingent robot more than a noncontingent one (Breazeal et al., 2016). Conversely, Tielman et al. (2014) found that a non-affective robot was perceived as more trustworthy than an affective robot. Therefore, affective experiences are crucial in the development and maintenance of trustworthy child–robot relationships. However, the aforementioned research showed that the results are somewhat inconsistent when evaluating the effects of the robot’s behaviors on children’s perception of trustworthiness.

As evidence suggests, children develop their trust models based on robot-related factors such as attribute factors—robot personality, expressiveness, embodiment, and anthropomorphism—and performance factors such as the robot’s behaviors (Bethel et al., 2016; Calvo et al., 2020; van Straten et al., 2020). However, it is yet to be understood which behaviors elicit higher social and competency trust in social robots, and how theories such as regulatory focus can be applied in the domain of child education.

In sum, RFT may be beneficial in cHRI, especially when the robot’s role is that of a companion for children. However, it is yet to be understood whether and how a robot that uses regulatory focus strategies affects children’s perceptions of the robot and the child–robot relationship formation. To the best of our knowledge, this is the first study in the literature that investigates the effects of regulatory focus design on child–robot affective relationship formation and children’s perception of the trustworthiness, likability, and competence of the robot.

## 3 Materials and Methods

### 3.1 Research Questions

There is evidence that children rely on the perceptions of competence and benevolence to determine whom to trust (Landrum et al., 2013). Besides, the robot’s behaviors have a significant impact on the development of competency trust, whereas the robot’s attributes affect social trust (van Straten et al., 2020). In this study, we aimed to investigate the effects of RFT on cHRI. The literature on virtual agents showed that prevention focus provoked lower ratings of perceived likability (Faur et al., 2015). However, it is yet to be understood how RFT influences children’s perception of a robot in terms of trust-related dimensions. Thus, we pose the following research question (RQ):

**RQ1:** Does regulatory focus influence children’s perception of a robot in terms of likability, competency, and trustworthiness?

Moreover, we wanted to explore the connections between children’s reliance on the robot’s suggestions and their perceptions of the robot’s trustworthiness during the activity. Studies in cHRI suggest that following the suggestions or recommendations of a robotic system is an objective measure used to capture children’s trust in robots (Groom et al., 2011; Geiskkovitch et al., 2019); hence, we pose the following research question:

**RQ2:** Does regulatory focus affect the way children follow the robot’s suggestions?

To address the aforementioned RQs, we designed a user study with Regulatory Focus as the between-subject factor with two conditions: prevention-focused and promotion-focused.

### 3.2 Participants

We conducted the study at two private, local, international schools in Lisbon, Portugal. A total of 69 children from the second and third grades (33 girls and 36 boys) took part in the study. They ranged in age from 7 to 9 years (M=7.58,SD=0.58). We excluded data from eight participants for reasons such as dropping the activity or speaking to the robot in a different language than English. After exclusion, 32 children (17 girls and 15 boys) were randomly assigned to the promotion-focused condition and 29 children (14 girls and 15 boys) were randomly assigned to the prevention-focused condition.

### 3.3 Apparatus and Stimuli

**FIGURE 1:**
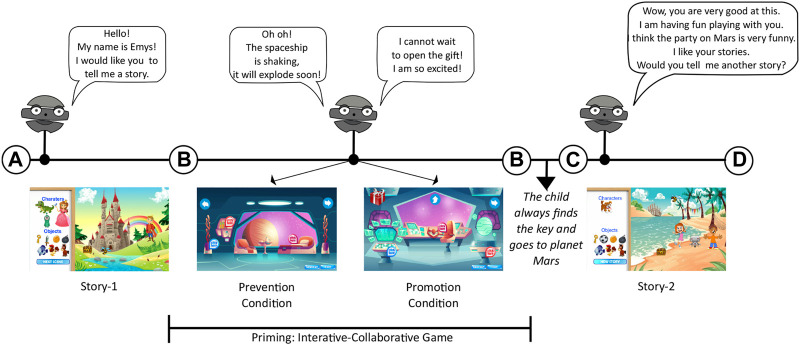
Flow of the interactive–collaborative activity. **(A)** Story-1, the child tells the first story to the robot. **(B)** Priming Game, the child and the robot play a goal-oriented game. The game has two versions: prevention and promotion. In both versions, the child always achieves the goal. **(C)** Story-2, the child tells a second story to the robot. **(D)** Questionnaires. Note: in this article, we only focus on the “priming” part of the activity.

We built an interactive–collaborative game to create a cHRI scenario. The game consisted of three parts: 1) interactive story-1, where the child was asked to tell a first story to the robot, 2) interactive–collaborative game using regulatory focus strategies, where the child was asked to reach a goal either with a prevention- or a promotion-focused robot, and 3) interactive story-2, where the child was asked to tell a second story to the robot. Figure 1 shows the flow of the overall activity.

For the purpose of this study, we focus only on *Priming: Interactive–Collaborative Game* (i.e., part two) out of the overall activity. We are solely interested in understanding the effect of regulatory focus design on trust perception in a goal-oriented activity. The effects of RFT on children’s learning are outside the scope of this study and are part of future work.

The game was created using Unity Game Engine^1^ for the graphical interface. As embodiment, we used the EMotive headY System (EMYS)^2^, a robotic head that consists of three metallic discs, equipped with a pair of eyes mounted on a movable neck. EMYS can convey different facial expressions for emotions. In a user study, children aged between 8 and 12 years validated the six basic emotions displayed by the robot (Kedzierski et al., 2013).

#### 3.3.1 Priming: Interactive–Collaborative Game

Priming is a technique used in research to elicit emotions (Neumann, 2000). In the promotion-focused condition, we were interested in eliciting feelings of excitement and happiness, whereas in the prevention-focused condition we paid attention to prompting feelings of anxiety and relief (Higgins, 1998; Baas et al., 2008). To accomplish this, we designed a collaborative game between the child and the robot. The game was designed in such a way that children could imagine themselves locked in a spaceship together with the robot. RFT design is oriented toward goal attainment; thus, the game was also goal-oriented. The child and the robot had a specific goal: *find the key to get out of the spaceship and go to planet Mars*. We built two versions of the game (Promotion and Prevention). In the prevention version, we focused on loss aversion: the motivation to achieve the goal, that is, to find the key, was to get out of the spaceship before it exploded. On the contrary, in the promotion version, the approach was toward reward seeking: if the goal was reached, participants received a gift.

The graphical interface consisted of three different scenes representing three rooms in the spaceship. Each room had a set of buttons the child could click on to get a hint or the key to get out. The hints and options were identical across conditions. The first and second rooms contained two buttons that did not have a hint or the key, one button with a hint, and two arrows which led to the same next room. The third room contained one button with neither a hint nor the key, one button with a hint, and one button with the key.

The robot’s verbal behaviors were designed to provide suggestions (e.g., “I think we should click on the arrow on the right”), to ask for requests (e.g., “Oh we have a message, can you read it for me?”), and to express emotions through verbal cues (e.g., “I am so scared of the explosion” and “I am so excited to see what is inside the gift”) and facial expressions (Kedzierski et al., 2013). The robot’s suggestions were identical across conditions, and they could be right or wrong suggestions (e.g., the robot could suggest clicking on a button that does not have a hint or the key). However, the robot’s emotions were intended to prime participants with a specific regulatory focus–related emotion (i.e., happiness vs. fear) and differed between conditions as described below:

*Promotion-Focused Robot:* The robot exhibited facial expressions of happiness and conveyed emotions through verbal messages such as “I am so excited to do this! I want to see what is inside our gift!,” “I cannot wait to open the gift! I am so excited!,” or “Wohoo! We are finally on planet Mars, I am so happy!”
*Prevention-Focused Robot:* The robot exhibited facial expressions of fear and conveyed emotions through verbal messages such as “I am so scared of the explosion! Let’s try to do this quickly!,” “Hurry up! We need to find the key before the spaceship explodes!,” and “We are finally on planet Mars, I feel so much better now!”

#### 3.3.2 Storytelling Activity

The storytelling activity consisted of two parts of the activity: story-1 and story-2 (e.g., pre- and post-test), see Figure 1. Each version of the story activity included four main characters, nine objects, and different scenario topics children could choose from to tell the story they wanted. Characters, objects, and scenario topics were different between the first and the second story to avoid repetitive stories. Two topics were designed for story-1 (e.g., park and castle) and three topics for story-2 (e.g., rainforest, beach, and farm). Also, the child could navigate through three different scenes for each topic. The robot’s behaviors consisted of a set of verbal behaviors to greet the participant (e.g., “Hello” and “What is your name?”), give instructions about the activity (e.g., “Select one story between the park and the castle by touching the button”), and encourage the child to tell or continue the story by asking questions (e.g., “And then what happens?”), providing feedback (e.g., “That’s a great choice. I like stories about princesses, princes and fantasy.”) or giving value to the story (e.g., “You are the best storyteller!”). For the storytelling activity, the robot’s behaviors were the same for story-1 and story-2. As part of our future work, we plan to measure the effects of regulatory focus design (Section 3.3.1) on narrative creativity.

### 3.4 Procedure

**FIGURE 2:**
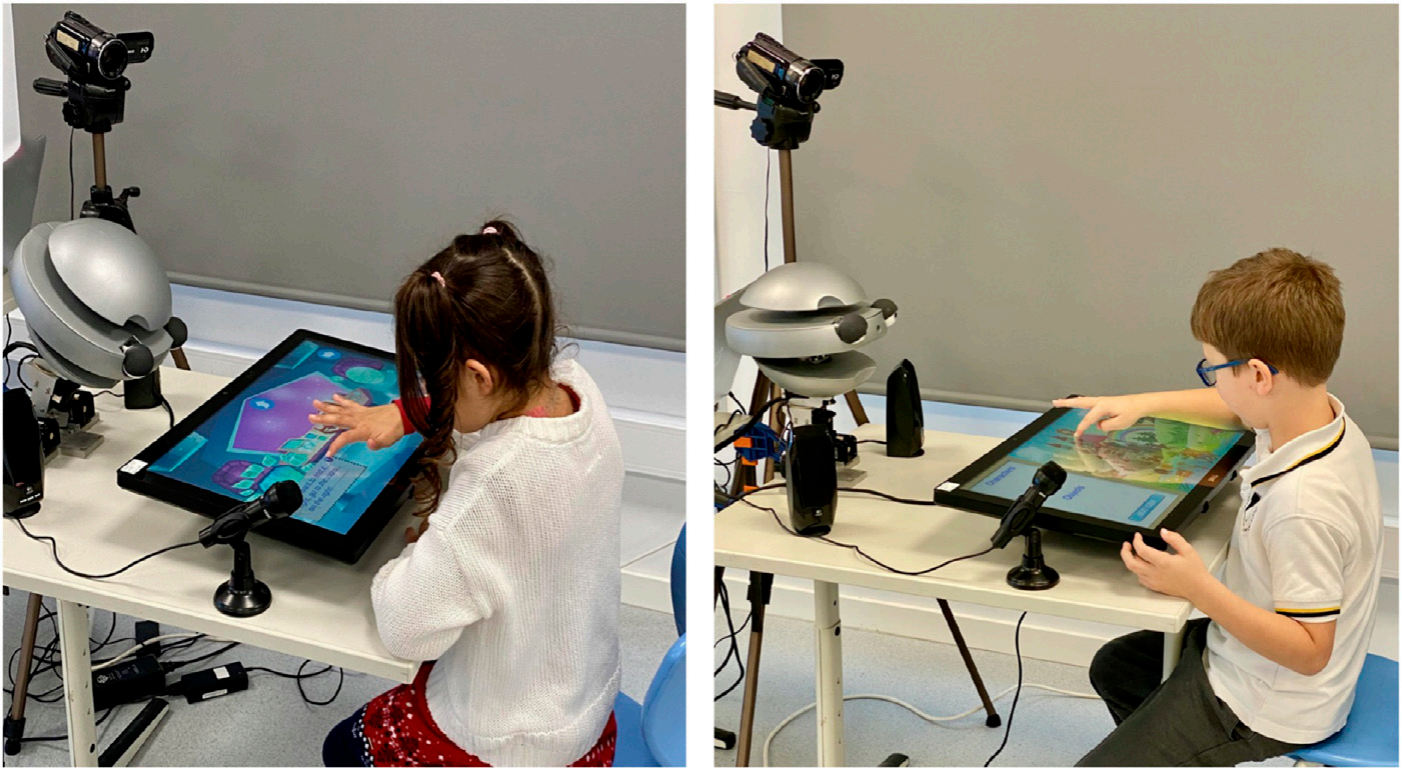
Children interacting with the robot during the interactive–collaborative activity.

The experiment took place at the children’s schools in an unused classroom. The robot was placed on a table facing the participant. The game was displayed on a touch screen between them. A microphone was placed in front of the child to record the audio data. We used two cameras to record video data. One was used to capture the frontal view with emphasis on the child’s face, while the other was used to capture the lateral view with emphasis on the child’s input to the touch screen (Figure 2). Participants were randomly assigned to one of the conditions. Two experimenters (A and B) were present in the room during the interaction. Experimenter A guided the child through the different stages of the activity, whereas experimenter B teleoperated the robot. Experimenter A started by greeting the child, introducing herself, and explaining the first part of the activity (Story-1). She instructed the child on how to use the interface on the touch screen to tell the story to the robot. Experimenter A told the child that they could tell the story they wanted without any time limit and asked the child to notify her when they had finished the story. Once the participant completed story-1, experimenter A explained the second part of the activity (priming) to them and asked the child to imagine themselves locked in a spaceship together with a robot. The experimenter explicitly told the child that if they managed to get out of the spaceship they would either receive a gift (promotion-focused condition) or avoid the explosion (prevention-focused condition). Once the child finished the game, experimenter A explained the third part of the activity (Story-2) and instructed the child to tell another story to the robot as in the first part, but using different characters, objects, and topics, and notify her when they had finished. Right after the interaction, experimenter A asked the child to fill in a questionnaire on a tablet. The questionnaire included measures of perceived trust, likability, enjoyment, and competence. After filling in the questionnaire, the experimenter debriefed the participants and thanked them for their participation.

### 3.5 Measures

As stated in Section 2.2, due to the multidimensional nature of trust in cHRI (i.e., social trust and competency trust), trust is captured by using different measures as children may use multiple sources of information to make judgments of trustworthiness (Bernath and Feshbach, 1995) (Carpenter and Nielsen, 2008). In our study, we used subjective (e.g., self-reports) and objective (e.g., children’s behavior) measures to assess children’s trust in robots (Table 1).

**TABLE 1:** Summary of subjective and objective measures and their association with trust dimensions.

Item measured	Code	Dependent variable	Type of measure	Trust dimension
Liking	QLik	Likability	Subjective	Relationship formation
Friendliness	QFri	Likability	Subjective	Relationship formation
Imagination	QIma	Competence	Subjective	Competency trust
Helpfulness	QHelp	Competence	Subjective	Competency trust
Advice	QAdv	Trust	Subjective	Competency trust
Follow-suggestions	QFolSug	Trust	Subjective	Competency trust
Tell-secrets	QSec	Trust	Subjective	Social trust
Compliance with the robot’s suggestions	MCSug	Trust	Objective	Competency trust
Resistance to the robot’s suggestions	MRSug	Trust	Objective	Competency trust
Compliance with the robot’s requests	MCReq	Trust	Objective	Competency trust
Resistance to the robot’s requests	MRReq	Trust	Objective	Competency trust
Free actions	MFAct	N.A.	Objective	N.A.

#### 3.5.1 Subjective Measures

Bernath and Feshbach stated that children’s perceptions of social trust are partially captured by social behavior measures (Bernath and Feshbach, 1995). Thus, we measured the robot’s likability in terms of liking and friendliness (Heerink et al., 2010; Straten et al., 2020). To investigate how children judge the perceived competence of the robot, we measured good imagination and helpfulness (Bernath and Feshbach, 1995). Moreover, we measured trust items to capture both social and competency trust. We took inspiration from the methods presented in the study by Heerink et al. (2010). We selected three items—tell-secrets, trust-advice, and follow-suggestions. We designed a questionnaire with the seven items presented in Table 2 measured on a 5-point Likert scale.

**TABLE 2:** Questionnaire for subjective measures.

Question	Code
I liked the robot Emys	QLik
I think the robot Emys was friendly	QFri
I think the robot Emys had a good imagination	QIma
The robot Emys helped me to create a better story	QHelp
I would trust the robot Emys if she gave me an advice	QAdv
I would follow the suggestions the robot Emys gives me	QFolSug
I would tell Emys my secrets	QSec

#### 3.5.2 Objective Measures

On one hand, Madsen and Gregor (2000) defined trust as the extent to which a user is confident in, and willing to act on the basis of, the recommendations, actions, and decisions of a system. On the other hand, Lee and See (2004) found that trust influences rely on automation. This suggests that children’s reliance on social robots might be guided by their perception of trustworthiness (Verhagen et al., 2019). To investigate the effects of regulatory focus design on children’s reliance on the robot’s recommendations, we defined five objective measures, as follows:

• Compliance-Suggestions (MCSug): Participant is in compliance with the robot’s suggestion.
• Resistance-Suggestions (MRSug): Participant does not accept the robot’s suggestion.
• Compliance-Request (MCReg): Participant is in compliance with the robot’s request.
• Resistance-Request (MRReq): Participant does not accept the robot’s request.
• Free-Action (MFAct): Participant is free to make any action. It means that the robot does not give suggestion nor makes a request.

We had to exclude further participants’ data for this analysis because of missing lateral videos. In total, 52 videos were analyzed for objective measures of trust (24 in the prevention-focused condition and 28 in the promotion-focused condition). We designed a coding scheme based on the child’s and the robot’s verbal behavior only in the interactive–collaborative game (priming). To validate the coding scheme, two researchers annotated the same portion (20%) of the video data. Hence, 11 videos were randomly selected to ensure proportional representation between experimental conditions. An inter-rater reliability analysis using Cohen’s Kappa statistic was performed to determine consistency among raters. The overall inter-rater agreement level across all items was 0.71 on average. Our results are in the range of substantial strength for agreement (Landis and Koch, 1977). Table 3 shows the inter-rater agreement for each item coded.

**TABLE 3:** Inter-rater agreement by item.

Objective measure	Cohen’s Kappa
Number of the robot’s suggestions	0.79
Number of the robot’s requests	0.62
Number of times children accept a suggestion	0.67
Number of times children do not accept a suggestion	0.75
Number of times children accept a request	0.87
Number of times children do not accept a request	0.62
Number of times children take a free action	0.65

We counted the number of times the participant accepted the robot’s suggestion and/or request with respect to the number of times the robot gave a suggestion and/or asked for a request. These were converted to percentages for ease of interpretation. Scores toward 100% mean that the children accepted most of the robot’s suggestions/requests. Conversely, scores near 0% mean that the children were reluctant toward the robot’s suggestions/requests.

## 4 Results

### 4.1 Manipulation Check

As the literature proposed, regulatory focus design triggers positive feelings (e.g., happiness and excitement) in promotion-focused self-regulation and negative feelings (e.g., stress and anxiety) in prevention-focused self-regulation (Higgins, 1998; Higgins and Cornwell, 2016). Moreover, Higgins and Cornwell (2016) showed that promotion- and prevention-focused self-regulation is associated with high and low social engagement, respectively. As expressions of stress are not easily measured from video analysis, to check if our manipulation worked, we opted for measuring differences in expressions of happiness and social engagement between the two conditions.

To examine if children were primed with happiness, we analyzed children’s smiles and facial expressions of joy. We used Affectiva^3^ software for facial expression analysis due to its accurate rates and robustness at extracting data (Garcia-Garcia et al., 2017). We used the Affectiva Javascript SDK to analyze the frontal camera videos. The Affectiva Javascript SDK uses deep learning algorithms for facial expression analysis. It detects seven emotions (anger, contempt, disgust, fear, joy, sadness, and surprise) and 15 expressions (including brow raise, brow furrow, cheek raise, smile, and smirk). The software generates a text file with values for each emotion and expression extracted in a range from 0 to 100 (i.e., from no expression detected to fully present). For the current analysis, we only included joy and smile.

A Wilcoxon signed-rank nonparametric test revealed that children show significantly more expressions of happiness in terms of smile (W=199,p=0.013,M=9.45,SD=12.92) and joy (W=216,p=0.03,M=7.52,SD=12.04) in the promotion-focused condition than in the prevention-focused condition (Elgarf et al., 2021).

Concerning engagement, we assessed engagement strength by using two measures of engagement: affective engagement, measured with the Affectiva SDK, and verbal engagement, measured through the child’s social verbal behavior toward the robot via annotated verbal behaviors from video data. The Affectiva SDK calculates engagement by computing emotional engagement based on facial muscle activation (e.g., brow raise, nose wrinkle, chain raise, etc.) and scores of sentiment valence that illustrate the user’s expressiveness. Nonparametric tests revealed a significant effect of regulatory focus design on both measures of engagement, affective engagement (p=.038,M=33.3,SD=18.84) and verbal engagement (W=236,p=.009,M=0.01,SD=0.01). Results suggest that children were more socially engaged in the promotion-focused condition than in the prevention-focused condition. Data analysis, procedures, and methods for the analysis of happiness and social engagement are explained in detail in the study by Elgarf et al. (2021).

Based on these results, we conclude that regulatory focused design was successfully implemented in the game. Thus, we continue with further analysis of the effect of RFT on trust perception.

### 4.2 Children’s Perception of Likability, Competence, and Trustworthiness

We ran a Kolmogorov–Smirnov test to check normality. All our dependent variables concerning subjective measures (Table 2) deviated significantly from normal. Therefore, we ran a Mann–Whitney test to analyze differences in the perception of likability, competence, and trustworthiness between conditions and investigate **RQ1**.

While it is likely that social-trust might be captured by relevant relationship formation constructs such as liking and friendliness (Bernath and Feshbach, 1995; Straten et al., 2020), we assessed the perceived likability of the robot in our analysis. We found a significant effect of regulatory focus on the likability of the robot. Concerning *QLik*, children rated the prevention-focused robot (M=4.93,SD=0.38) as more likeable than the promotion-focused robot (M=4.66,SD=0.67), $U\left(N_{Prom}=29,N_{Prev}=27\right)=482,z=-2.32,p=.020,r=-.31$. Moreover, results did not reveal any significant effect of regulatory focus on perceived friendliness (*QFri*) $U\left(N_{Prom}=28,N_{Prev}=27\right)=406,z=.87,p=.383,r=.12$.

Concerning perceived competence, we did not find any significant effect of regulatory focus on the dependent variables, *QIma* ($U\left(N_{Prom}=29,N_{Prev}=27\right)=360,z=-.58,p=.561,r=-.08$) and *QHelp* ($U\left(N_{Prom}=20,N_{Prev}=16\right)=186,z=.79,p=.430,r=.13$). To assess children’s perceived trustworthiness of the robot, we analyzed the corresponding subjective measures or items of trust. Again, we did not find any significant effect of regulatory focus on the dependent variables, *QAdv* ($U\left(N_{Prom}=28,N_{Prev}=26\right)=363,z=-.02,p=.984,r=-.01$), *QFolSug* ($U\left(N_{Prom}=29,N_{Prev}=26\right)=379,z=.04,p=.969,r=.01$), and *QSec* ($U\left(N_{Prom}=28,N_{Prev}=27\right)=417,z=.68,p=.496,r=.09$).

Other studies focused on assessing trust in social robots have suggested that children’s perception of trust in a robot is rather inferred from initial impressions of competence and likability (Calvo-Barajas et al., 2020).

We ran Spearman’s rank correlation analysis to examine if likability and competence were positively or negatively correlated with trust. The results are summarized in Table 4. The results revealed a positive significant correlation between the items of likability (QLik and QFri), competence (QIma and QHelp), and trust (QAvd and QFolSug). We found that the trust item QSec was significantly positively correlated with the items evaluated for perceiving competence (QIma and QHelp). This exploratory analysis shows that children’s perception of the robot’s likability and competence positively impacts participants’ trust in the robot.

**TABLE 4:** Spearman’s rank correlations of likability and competence with trust.

	Likability		Competence	
Trust	Liking	Friendliness	Imagination	Helpfulness
Trust-advice	0.54^a^	0.47^a^	0.46^b^	0.38^c^
Follow-suggestions	0.28^c^	0.32^c^	0.47^a^	0.31
Tell-secrets	0.22	0.14	0.34^c^	0.47^b^

a *p* < 0.001.

b *p* < 0.01.

c *p* < 0.05.

### 4.3 Children’s Following of the Robot’s Suggestions

**RQ2** aimed to investigate the effect of regulatory focus design on children’s acceptance of the robot’s suggestions. To accomplish this, we defined five dependent variables, MCSug, MRSug, MCReq, MRReq, and MFAct, described in Section 3.5.2. To understand the effect of the condition on the dependent variables, the dependent variables were measured as frequencies rather than averages. In other words, we counted the number of times the child accepted the robot’s suggestions. These measures were transformed into percentages for easier interpretation.

**FIGURE 3:**
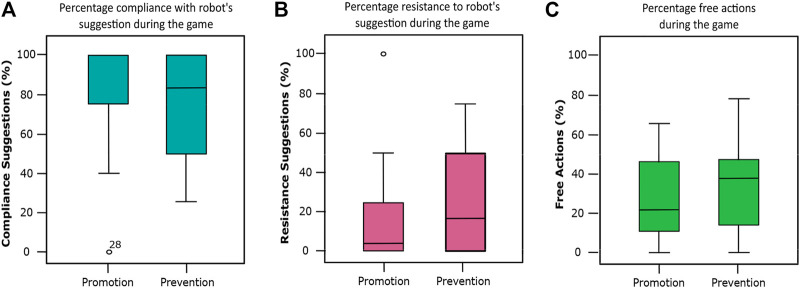
**(A)** is the percentage of compliance suggestions, **(B)** is the resistance suggestions, and **(C)** is the free actions per condition during the interactive–collaborative game. There were no significant differences between conditions.

We ran a Kolmogorov–Smirnov test to check normality. All our dependent variables deviated significantly from normal. Thus, we ran a Mann–Whitney *U* test. The analysis did not reveal any significant difference between the two conditions for *MCSug* ($U\left(N_{Prom}=28,N_{Prev}=23\right)=254,z=-1.34,p=.179,r=-.19$), *MRSug*($U\left(N_{Prom}=28,N_{Prev}=23\right)=385,z=1.26,p=.209,r=.17$), *MCReq*($U\left(N_{Prom}=28,N_{Prev}=24\right)=355,z=.49,p=.627,r=.07$), *MRReq*($U\left(N_{Prom}=28,N_{Prev}=24\right)=316,z=-.47,p=.627,r=-.07$), *MFAct*($U\left(N_{Prom}=28,N_{Prev}=24\right)=388,z=.96,p=.339,r=.14$). Figure 3 shows the distribution of children’s acceptance of and resistance to the robot’s suggestions, as well as free actions. There was no significant difference between the conditions.

As an exploratory analysis, we investigated the relationship between subjective measures (i.e., QLik, QFri, QIma, QHelp, QAdv, QFolSug, and QSec) and objective measures (i.e., MCSug, MRSug, MCReq, MRReq, and MFAct). To do so, we ran Spearman’s rank correlation analysis. The results are summarized in Table 5. We found that the perceived competence of the robot in terms of helpfulness was significantly negatively correlated with children’s acceptance rate of the robot’s suggestions. Conversely, children’s perception of the robot’s helpfulness significantly impacted children’s resistance to following the robot’s suggestions.

**TABLE 5:** Spearman’s rank correlations of subjective measures with objective measures.

	Subjective measures						
	Likability		Competency		Trust		
Objective measures	Liking	Friendliness	Imagination	Helpfulness	Trust-advice	Follow-suggestions	Secrets
Compliance with suggestions	−0.20	0.04	0.02	−0.39^a^	0.20	0.21	−0.09
Resistance to suggestions	0.21	−0.04	−0.01	0.43^a^	−0.19	−0.21	0.13
Compliance with requests	0.10	−0.02	−0.02	0.17	0.14	0.05	0.03
Resistance to requests	−0.10	0.02	0.02	−0.17	−0.14	−0.05	−0.03
Free actions	0.06	0.05	−0.13	0.23	−0.05	−0.06	−0.05

a *p* < 0.05.

b *p* < 0.01.

## 5 Discussion

### 5.1 Effect of RFT on Perceived Likability, Competence, and Trustworthiness (RQ1)

We found that a regulatory focus design scenario affects children’s perceptions of the likability of the robot. Our results suggest that children who interact with a social robot in a goal-oriented activity liked the robot more when it motivated them to achieve a goal to avoid risk (prevention-focused condition) than when it motivated them to get a reward (promotion-focused condition). This result is in line with previous work with virtual agents that found that prevention focus positively affects the likability of a virtual agent for users (Faur et al., 2015). One possible interpretation of these results is that the prevention-focused robot expressed verbal behaviors that communicated that it was scared of the explosion (Section 3.3.1) and, as a consequence, children might have associated these behaviors with a robot’s vulnerability, leading to an increased perception of the likability of the robot. Prior work has found that vulnerable disclosures may drive more feelings of companionship with a robot in teenagers (Martelaro et al., 2016).

An interesting point of discussion concerns the relation between children’s perception of the robot’s likability measured post-interaction and their behavior during the priming game. While children rated the promotion robot as less likeable, behavioral data based on the facial expressions of emotion and engagement (see Section 4.1) showed that the promotion robot evoked more happiness and social engagement in children. However, this is not surprising as it is well established in social psychology that different types of measurement elicit different responses, and these different responses often do not correlate (Greenwald, 1990; De Houwer and Moors, 2007). It addresses an open question on the methods used to measure children’s perceptions of and their social interaction with social robots. This is crucial when investigating child–robot relationship formation, as it has been shown that the type of measure (subjective vs. objective) influences how children interpret their social trust in and liking for a social robot (Stower et al., 2021).

Moreover, the results showed no significant difference in evaluations of perceived competence and trustworthiness for any of the items measured. This suggests that a regulatory focus design scenario does not directly affect the way children create a trust model of the robot. One possible explanation of this result could be that the robot’s performance (equally for both conditions) had a stronger effect on children’s perception of trust and competence with regard to the robot than the robot’s expressiveness (i.e., happiness vs. fear), as responsiveness is associated with children’s trust in a robot (van Straten et al., 2020). However, further investigation is needed to support this assertion.

Correlation analyses suggest that children’s trust in a robot might be captured by impressions of likability and competence that the robot evokes. This result is in line with previous studies that suggest that these constructs are predictors of trust (Calvo-Barajas et al., 2020). In particular, we found that likability and competence positively affect the perception of competency-trust. This finding is surprising, because the literature has shown that relationship formation constructs (e.g., likability and friendliness) overlap with social-trust (Bernath and Feshbach, 1995; Stower et al., 2021; van Straten et al., 2020). In contrast, we found that the perceived competence the robot elicits has a positive effect on the children’s consent to disclose their secrets to the robot. Again, this result is unexpected as measures of self-disclosure, keep-, and tell-secrets are associated with the definition of social-trust (i.e., “belief that the robot will keep its word or promises”) (Stower et al., 2021).

We presume that a regulatory focus design scenario influences the way children build their trust model of a social robot, where the perceived likability and competence are positively significantly correlated with trust. One possible argument could be that in the prevention-focused condition, children experienced the need for security to reduce risk. Thus, when they accomplished the goal (i.e., getting out of the spaceship before it explodes), their perception of the helpfulness of the robot at avoiding a specific threat might have increased their social-trust in the robot. This preliminary explanation could be linked to the fact that prevention-focused behaviors are mediated by privacy concerns in adults (Wirtz and Lwin, 2009). Since our study is the first study of its nature, we need more evidence to support this claim.

Nevertheless, the conceptualization and operationalization of trust are challenging, especially in cHRI, as its definition differs among individuals. Hence, we considered the multifaceted property of trust as a key element to be exploited for a better understanding of how children make judgments of trustworthiness. The design of tailored methods and measures to capture children’s trust in robots is gaining the attention of researchers from different fields to reduce the heterogeneity of this construct among studies (van Straten et al., 2020). We hope that our findings provide insights that can be used to build on the conceptualization of children’s trust and its implications with regard to the relationship with a robot.

### 5.2 Effect of RFT on Children’s Following of the Robot’s Suggestions (RQ2)

Concerning children’s willingness to accept or resist the robot’s suggestions, we found that a regulatory focus design scenario does not significantly affect children’s rates of acceptance of the robot’s suggestions and requests. Even though we did not find any significant difference, we noticed that on average the prevention-focused condition elicits higher resistance in children to following the robot’s suggestions, whereas the promotion-focused condition seems to influence a higher reliance on the robot’s suggestions, which is associated with higher competency trust in the robot. This preliminary result aligns with prior research in psychology, as it suggests that promotion-focused individuals are open to new ideas and experiences (Friedman and Förster, 2001). However, our results were not statistically significant, and more investigation is needed to claim this statement. Nevertheless, we believe that our findings could be beneficial for further studies as they provide new insights into the design of the robot’s affective behaviors based on RFT to elicit positive emotions, a paradigm that has not been studied before in cHRI. Therefore, it could be beneficial for child–robot relationship formation, especially in the domain of child–robot educational interactions.

Moreover, we find the ceiling effect observed in children’s compliance with the robot’s suggestions interesting but not surprising. In several cases, children accepted all the suggestions, taking into account that some of them were wrong. These results raise opportunities, but also concerns, regarding the use of social robots as learning companions for young children, as has been presented in prior work (Vollmer et al., 2018).

Finally, the exploratory analysis revealed that the perceived helpfulness of the robot negatively impacted the children’s compliance with the robot’s suggestions. This result is confounding, as we would have expected that children who perceived the robot to be more helpful would be more likely to follow the robot’s suggestions. However, this result should be interpreted with caution, as helpfulness was assessed as a subjective post-interaction measure, whereas children’s compliance/resistance with/to the robot’s suggestions was assessed as an objective measure during the interaction. On one hand, we presume that other parts of the activity could have influenced children’s judgment of the robot’s helpfulness. On the other hand, previous research studies have found that subjective and objective measures elicited different responses when evaluating children’s competency trust in a social robot (Stower et al., 2021).

Overall, our results suggested that objective measures are not always positively correlated with subjective measures. However, as indicated in the study by Belpaeme et al. (2013), it is crucial to validate if the desired outcome is captured by the proposed objective measure, as some constructs are harder to measure than others. To explore the relationship between subjective and objective measures, we would like to further investigate whether “following the robot’s suggestions” is an appropriate and reliable measure to capture children’s competency trust in the robot in a regulatory focus priming scenario without having the storytelling activity component, as proposed in our pilot study Calvo-Barajas et al. (2021).

### 5.3 Limitations and Future Work

One of the limitations of this study is that we were not able to explore the effects of a regulatory focus design scenario on children’s compliance with right and wrong suggestions. The nature of the interaction did not allow us to have the same amount of right and wrong suggestions between conditions. However, the exploration of these effects would be an interesting topic for future research. As such, we aim at increasing the number of times children have to comply with or resist the robot’s suggestion, and this might also improve the inter-rater agreement score.

Another limitation is that we could not fully explore whether the manipulation of the regulatory focus induced negative feelings of stress in the participants. We were only able to measure differences in terms of expressions of happiness and social engagement between the two conditions. In future work, the measurement of electrodermal activity (EDA) could be considered to analyze children’s stress level.

In this study, we were interested in investigating regulatory focus theory as a priming strategy rather than exploring matching as the regulatory fit theory suggests. Therefore, we did not assess children’s regulatory orientation. Nevertheless, we believe that this is an interesting topic for further investigation, as individual differences might influence children’s social interactions and relationship with robots (Shahid et al., 2014). In addition, it would be worthwhile to explore different methods of the robot’s adaptation in cHRI.

As we discussed before, we did not find any significant difference in regulatory focus design on child–robot relationship formation. To provide more insights into the implementation of RFT as a technique to be used in cHRI in an educational context, it would be interesting to study whether the robot’s presence influences the way children interact in a goal-oriented task based on RFT by introducing a control condition.

Finally, a parallel line of investigation, but outside the scope of this article, includes the exploration of whether and how RFT affects creativity performance in interactive storytelling. To do so, we aim to evaluate narrative creativity measures in children’s stories before and after the priming activity.

## 6 Conclusion

In this article, we presented a novel user study investigating the effects of a regulatory focus design scenario on children’s perception of likability, competence, and trustworthiness of a social robot. Besides, we evaluated the effect of a regulatory focus design scenario on children’s compliance with the robot’s suggestions.

We found that a regulatory focus design scenario significantly affected children’s perception of the likability of the robot, while perceived competence and trustworthiness did not change between conditions. Similarly, the motivation to achieve a goal did not significantly affect the way children followed the robot’s suggestions. Nevertheless, on average, the prevention-focused robot increased children’s resistance to following suggestions. Interestingly, the items used to capture children’s trust in a robot are correlated among them, suggesting that trust may be inferred by constructs of social cognition and social learning.

These findings are relevant to the study of trust in cHRI, as they provide new evidence on the effect of strategies based on RFT on perceived trust in a robot in an educational scenario, and they highlight the relevance of the multidimensional nature of trust when evaluating children’s judgments of trustworthiness of a social robot.

## Data Availability

The raw data supporting the conclusions of this article will be made available by the authors, without undue reservation.

## Footnotes

1
https://unity.com

2
https://robots.ieee.org/robots/emys/

3
https://www.affectiva.com
